# Revealing alterations in heart rate fluctuations during the progression of Chagas disease

**DOI:** 10.3389/fmed.2024.1438077

**Published:** 2024-09-10

**Authors:** Magdalena M. Defeo, Laura A. Delaplace, Juan C. Goin, Carina Tersigni, Leopoldo Garavaglia, Isabel M. Irurzun

**Affiliations:** ^1^Hospital Interzonal General de Agudos “Prof. R. Rossi”, La Plata, Argentina; ^2^Laboratorio de Salud Pública, Facultad de Ciencias Exactas, Universidad Nacional de La Plata, La Plata, Argentina; ^3^Centro de Estudios Farmacológicos y Botánicos (CEFyBO-CONICET-UBA) and II Cátedra de Farmacología, Facultad de Medicina, Universidad de Buenos Aires, Buenos Aires, Argentina; ^4^Centro de Investigaciones Opticas (CIOp-CCT La Plata. CONICET), La Plata, Argentina; ^5^Centro de Simulación Computacional para Aplicaciones Tecnológicas-Consejo Nacional de Investigaciones Científicas y Técnicas (CSC-CONICET), Buenos Aires, Argentina

**Keywords:** Chagas disease, heart rate variability, sinoatrial node, autonomic nervous system, fibrosis

## Abstract

**Introduction:**

The heart rate variability (HRV) continually evolves throughout life, reflecting modifications in the architecture of the sinoatrial node (SAN) and in the regulation of heart rate by the autonomic nervous system (ANS). Both can be considerably affected by Chagas disease, causing important changes in the complex nature of HRV. We aim to evaluate the ability of an index based on the false nearest neighbors method (FN10) to reflect these changes during disease progression.

**Methods:**

We perform a retrospective, descriptive, and cross-sectional study analyzing HRV time series of participants with Chagas disease. We determine the dependence of FN10 on age and sex in a healthy population, and then evaluate FN10 in individuals with Chagas disease.

**Results and discussion:**

In the healthy population, FN10 has a scaling behavior with age, which is independent of sex. In Chagas disease, some individuals show FN10 values significantly above those seen in the healthy population. We relate the findings to the pathophysiological mechanisms that determine the progression of the disease. The results indicate that FN10 may be a candidate prognostic biomarker for heart disease.

## 1 Introduction

Chagas disease (CD) is a chronic neglected tropical disease caused by the protozoan *Trypanosoma cruzi* that affects six–seven million people worldwide and causes around 12,000 deaths annually. CD is endemic in 21 countries and responsible for the highest burden among other parasitic diseases within the American continent. Yet, it has also become a concern in non-endemic countries of the Americas and the rest of the world due to human migration (1).

Soon after infection, individuals enter a two-month acute phase, which is characterized by high parasitemia and usually absent or mild symptoms. In the chronic phase, individuals with infection exhibit seropositive diagnostic tests against *T. cruzi* while parasites are rarely detected in circulation. Several years after infection 20%–30% of the individuals develop heart disease while up to 10% suffer from digestive and/or neural disorders.

Chronic Chagas heart disease may initially be clinically silent, but then can progress to conduction disturbances, ventricular arrhythmias, and dilated cardiomyopathy with heart failure, and other systemic or pulmonary embolisms (2). It is associated with high mortality and a plethora of severe clinical manifestations. However, neither the appearance of cardiac manifestations nor the individual pattern of heart damage can be predicted, and it evolves with variable prognosis among individuals (3).

Heart rate variability (HRV) quantifies the fluctuations in the duration between heartbeats, and is used in several diseases that affect the neural control of cardiovascular function as a prognostic index as well as a biomarker for treatment outcomes (4–19). It is a complex, scale-invariant signal whose characteristics in healthy people are predominantly determined by the autonomic nervous system (ANS) and the sinoatrial node (SAN) (20–22). Power spectral analysis has been used to evaluate the influence of both on the HRV. The spectrum is divided into three bands: a high frequency (HF), a low frequency (LF), and a very low frequency (VLF) band, assuming that the mechanisms that determine the HRV have signatures or characteristic frequencies, and that the variations among individuals in the population cause their dispersion to form bands. The main contribution of SAN is in the VLF band. The LF band is mainly attributed to the action of the sympathetic branch of the ANS, and the HF to the contribution of both the parasympathetic branch of the ANS and respiratory sinus arrhythmia (23, 24).

Another approach considers the HRV as a correlated signal with defined scaling factors in the time (α) or frequency (β) domain (25–31). The VLF, LF, and HF indices, as well as the α and β scale factors, among other measurements, have been calculated in the study of various pathologies such as diabetes, hypertension, heart failure, and heart diseases. Altered indices are observed in ventricular arrhythmias, heart failure, and after myocardial infarction.

Autonomic involvement is well established in advanced Chagas cardiomyopathy. Characteristic depletion of parasympathetic neurons in the heart, esophagus, and colon can be found in individuals with *T. cruzi* infection. Similar neuronal depopulation occurs in other cardiomyopathies, but in Chagas cardiomyopathy the reduction is more severe and extensive. Many individuals with CD present alterations in parasympathetic cardiac control even in the early stages of the disease before developing myocardial dysfunction (2, 32, 33). However, vagal denervation is not correlated with the severity of left ventricular (LV) dysfunction, and sympathetic denervation has also been detected in many individuals with CD (34–37). Diffuse and widely dispersed cardiac fibrosis affecting both the myocardium and the conduction system is a notable finding, which has been reported even in the early stages of chronic disease (38, 39).

An autoimmune response triggered by *T. cruzi* and mediated by molecular mimicry has also been proposed as a potential etiopathogenic mechanism for cardiac damage in chronic CD (40). Several studies have reported a high prevalence of anti-β_1_/β_2_ adrenergic receptor antibodies (anti-β_1, 2_AR Ab) in individuals with chronic CD with ventricular arrhythmias (34), while anti-*M*_2_-muscarinic-receptor antibodies (anti-*M*_2_R Ab) have been detected in those individuals with cardiac dysautonomia (35, 41, 42), sinus node dysfunction (34), and increased ventricular repolarization heterogeneity (43).

The prognostic value of anti-neurotransmitter receptor autoantibodies has also been tested. Within the chronic population with *T. cruzi* infection, nearly 30% of asymptomatic individuals are more likely to develop cardiac manifestations. Given that 29% of asymptomatic individuals and 98% of individuals with cardiomyopathy have been found to be seropositive for anti-β_1_AR Ab combined with anti-*M*_2_R Ab, seropositive individuals without symptoms are likely at risk of developing cardiomyopathy in the future (44).

The importance of developing risk stratification scores for individuals with chronic CD is recognized (2, 45–47). The Rassi score, for individuals with established cardiomyopathy, weighs the risk of 10-year mortality. The Sousa score is used to predict the risk of sudden cardiac death. Other predictors exist but are less reliable. The BENEFIT trial provided multinational risk data on individuals with mild to moderate Chagas cardiomyopathy.

Based on previous contributions we analyze the mechanisms that regulate heart rate and its fluctuations to envision a biomarker of disease progression.

In a recent study of HRV in healthy population we revealed new findings about the participation of the SAN and the ANS (48): (a) the ANS provides a broadband signal of constant power that appears at birth; (b) in the range of ages considered in this work, the contribution of the ANS decreases slightly with aging; (c) the SAN imposes a scaling behavior that starts at low frequencies and evolves continuously even in fetuses from the fifth month of gestation; (d) there is a progressive establishment of this scale behavior that reaches its maximum expression around the age of 7 (occupying the entire frequency range), remains stable until adulthood, and then recedes again toward low frequencies.

Separating the effects of the two contributions (ANS and SAN) is difficult, even in the frequency domain. This means that the approaches used until now, although essentially correct, are too simplistic when it comes to analyzing an evolutionary disease like Chagas. Rather, HRV should be considered as a signal having a continuous power spectrum that evolves smoothly with age. In this work, we consider that the complex nature of HRV can be revealed in a multidimensional space. This approach was previously used in individuals with ventricular arrhythmia and congestive heart failure (49–51).

We define an index called FN10, which depends on the age of healthy individuals, and we determine this dependence in individuals over 5 years of age. At that age, the architecture of the SAN has almost completely matured and the contribution of the ANS has reached its maximum power and begins to decrease. Then we evaluate the performance of FN10 in individuals with chronic Chagas disease across its different stages, considering age as a variable.

## 2 Materials and methods

We perform a retrospective, descriptive, and cross-sectional study analyzing a total of 340 HRV time series; 259 of them correspond to participants with Chagas disease and 81 series are of healthy donors. All data were taken before the COVID-19 pandemic.

The healthy population was selected from that used in Garavaglia et al. (22), according to the ages of the individuals. They were aged between 5 and 74 years, and 50% were women. Of these 81 individuals, 40 were enrolled as volunteers for our study and were aged between 5 and 55 years. Their HRV time series are available on PhysioNet: the research resource for complex physiological signals (52). Another 13 time series correspond to the MIT-BIH Normal Sinus Rhythm Database (53, 54), and 28 to the Normal Sinus Rhythm Database (55). These series were used to control the quality of our series since all data were acquired, examined, and corrected similarly, as we explain below (49–51). For this reason, they are also included in this study. Participants ranged in age from 20 to 74 years, 50% being women.

Individuals with Chagas disease (CD) were enrolled in areas of endemicity in Argentina (provinces of Salta, Jujuy, Chaco, and Santiago del Estero) and Bolivia (Department of Santa Cruz). Participants ranged in age from 12 to 86 years, 50% being women. Diagnosis of CD was confirmed based on two positive standard serological reactions against *T. cruzi*. All participants underwent clinical and cardiological evaluations, including standard 12-lead ECG, 24-h Holter ECG, and 2-D echocardiography. The 12-lead ECG was acquired after the clinical examination and before performing the Holter recording. Echocardiography was performed within 12 months after Holter recording. Based on an in-person questionnaire during the initial clinical examination, individuals who were pregnant or had thyroid disorders, diabetes, kidney disease, malignancies, or neurological disorders were not selected for the study, as well as individuals with chronic systemic diseases, any infectious disease other than CD, or previous antiparasitic treatment for *T. cruzi* infection. Participants with uncontrolled hypertension on clinical examination were not included in the study.

The data obtained from healthy population were used as control group (CG). Within the chronic population with *T. cruzi* infection, the data were classified according to the symptoms of the individuals as follows: Group A (GA) included individuals with neither electrocardiographic nor echocardiographic findings (*n* = 192); Group B (GB) included individuals with heart disease who had only electrocardiographic abnormalities (ECG or Holter) without echocardiographic findings (*n* = 48), and Group C (GC) included individuals with abnormal echocardiographic parameters, such as left ventricular dimension, systolic function, global or regional wall motion, or left ventricular ejection fraction, and with or without electrical findings (*n* = 19). Within GA, we did not include individuals on medication, while in GB and GC we excluded individuals who were taking autonomic agonist/antagonist drugs or psychotropics.

Holter monitoring was recorded for 24 h with digital three-channel DMS300 7 and DMS300 3A recorders, using 3M electrodes. The DMS recorders had a sampling rate of 1,024 Hz per channel for signal-averaged electrocardiography (SAEG) analysis, a read-in sampling rate of 512 Hz, and a write-out sampling rate of 128 Hz in the other cases. The signals were analyzed with CardioScan 10.0 and 11.0 software. The error in the RR interval determination was about 8 ms (twice the error in the determination of the R peak). The records of cardiac events were automatically detected and classified by the Holter software, and then examined and corrected by two cardiologists. We used the quality criteria established in previous studies (49), for all the time series used in this work, and the age was considered a continuous variable (22).

In this work, the complex nature of the HRV is evaluated by unfolding its phase space. A time series can be considered as the sequence of observations of a measurement function of a dynamic system evolving in a multidimensional phase space. The false nearest neighbor (FNN) method was proposed by Kennel, Brown and Abarbanel to display the phase space, and we used it before to determine an index called FN10 and analyze differences between healthy adults, individuals with supraventricular and ventricular arrhythmias, and those with congestive heart failure (49, 50, 56, 57). To calculate FN10 for each time series, we normalize it to zero mean and standard deviation of one and construct the FNN curve using the routine provided by the TISEAN (time series analysis) software package with the parameters *f* = 10 and τ = 1 (56, 58). The robustness of the method was evaluated in previous studies (49, 50). We used all the data available in each series (approximately varying between 54,000 and 120,000) and verified that our results were independent of the duration of the time series. This condition was met with a series length >20,000 beats, depending roughly on the embedding dimension. We also did a surrogate analysis and evaluated different values of the parameters. The FNN method provides the residual of false neighbors at a given spatial dimension (d) and FN10 is the FNN value at dimension 10, which is the highest embedding dimension value in healthy adults (49, 50). FN10 is age-dependent, and in the Results section a scale relationship is revealed by applying a logarithmic transformation to both the independent and dependent variables. The scale factor is determined by performing a linear regression fit using the least squares method. The size effects can be evaluated either through Cohen's *d* index or the regression coefficient (R). We used R in this work and the values obtained indicate that the size effects are negligible.

## 3 Results

**Figure 1:**
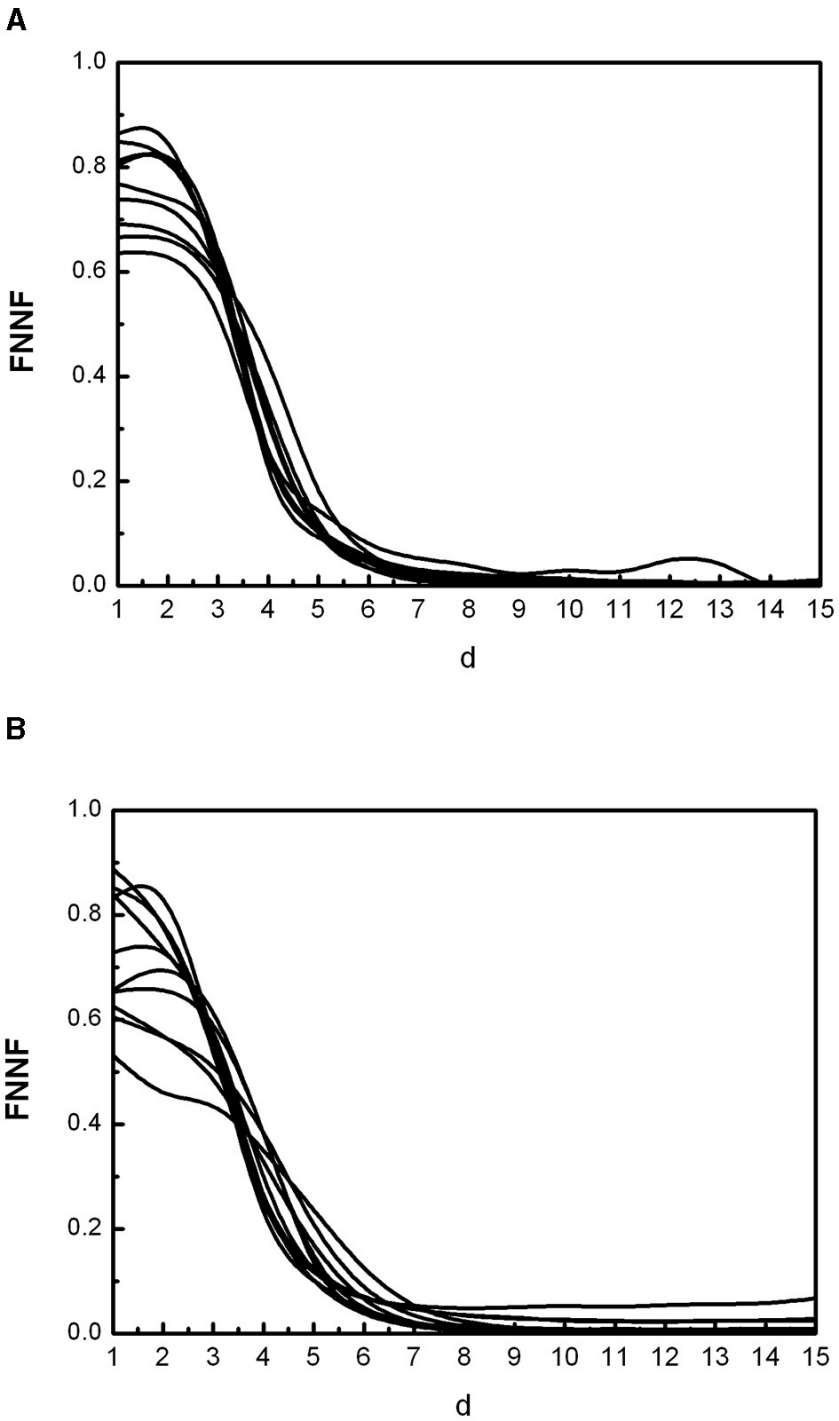
Some curves of false nearest neighbors (FNN) as a function of the spatial dimension (d) representative of the population of healthy individuals **(A)** and individuals with Chagas disease **(B)**.

Figure 1 shows some FNN curves for healthy adults (a) and individuals with CD (b). All the curves for healthy individuals are similar, with a fraction of false nearest neighbors decreasing monotonically as the dimension (d) of the phase space increases, until stabilizing at the so-called embedding dimension (*d*_0_). This behavior is the same in all healthy individuals over 7 years of age (49). The FNN curves for individuals with CD are different, indicating altered cardiac dynamics. One way to quantify the departure from normality is by measuring the fraction of false nearest neighbors at *d*_0_ = 10, which is the maximum embedding dimension determined in healthy individuals over 7 years; we call this value FN10. Note that we are not analyzing the shape of the curves for low *d* values because the phase space is still highly folded. Our goal is to evaluate the performance of FN10 in Chagas disease.

**Figure 2:**
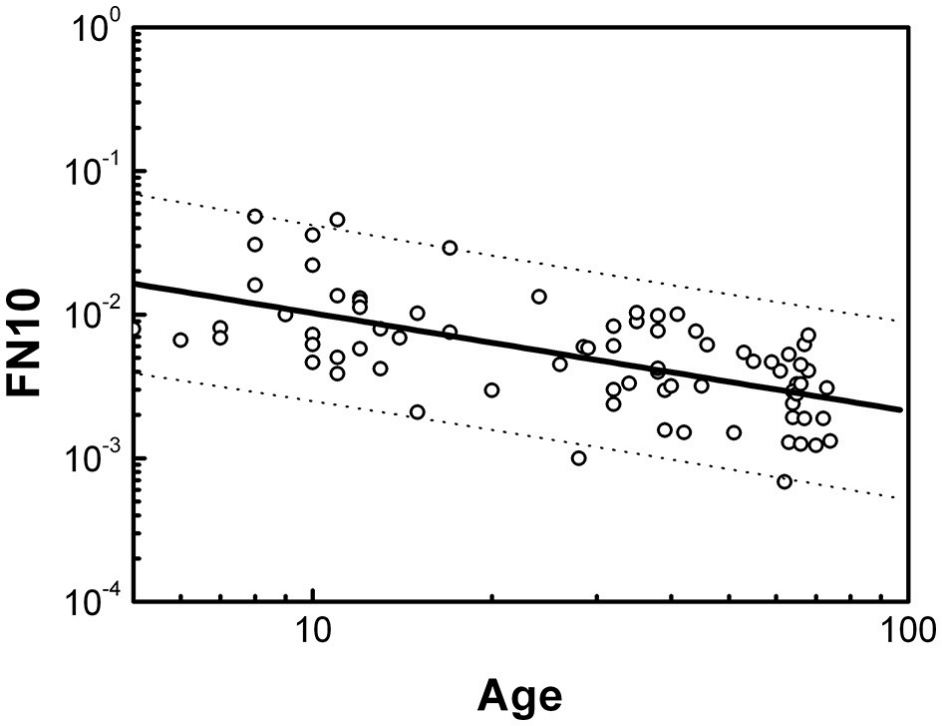
Dependence of FN10 on age expressed in years for the healthy population. Dotted lines define the boundaries of the prediction intervals with 95% confidence.

Figure 2 shows the dependence of FN10 on age in the healthy population. It can be seen that FN10 follows a scaling law given by


(1) $$\begin{array}{l} FN10=\left(0.049\pm 0.007\right)Age^{-\left(0.7\pm 0.1\right)} \end{array}$$

The statistical analysis is presented in Table 1. The size effects evaluated with the regression coefficient R are negligible. We did not find significant differences by sex. The dotted lines in Figure 2 indicate the prediction intervals with 95% confidence. A prediction interval is an estimate of a range of values over which future observations will occur with a certain probability, given what has already been observed. Thus, the FN10 value of a healthy individual should evolve throughout life within the prediction intervals, and we will consider the FN10 values that are within these intervals as normal (or negative).

**Table 1:** ANOVA table for linear regressions after logarithmic transformation in healthy population.

**Parameter**	**Value**	**Error**	**Statistic**		
**The entire population**					
A	–1.31	0.15	<0.0001		
B	–0.68	0.10	<0.0001		
R	0.62				
**Source**	**Degrees of freedom**	**Sum of squares**	**Mean squares**	*F*	**Statistic**
Model	1	4.265130820849	4.265130820849	46.600231773735	<0.0001
Error	79	7.230550622217	0.091525957243		
Total	80	11.4956888307			
**Females**					
A	–1.28	0.26	<0.0001		
B	–0.70	0.17	<0.0001		
R	0.59				
**Source**	**Degrees of freedom**	**Sum of squares**	**Mean squares**	*F*	**Statistic**
Model	1	1.560976408496	1.560976408496	16.907631172959	<0.0003
Error	40	3.87759872959	0.092323779276		
Total	41	5.44736281455			
**Males**					
A	–1.22	0.23	<0.0001		
B	–0.74	0.15	<0.0001		
R	0.67				
**Source**	**Degrees of freedom**	**Sum of squares**	**Mean squares**	*F*	**Statistic**
Model	1	1.951108182106	1.951108182106	24.11669595675	<0.0001
Error	37	2.993403527073	0.080902798029		
Total	38	4.944585348133			

Model: *y* = *A*+*Bx*.

**Figure 3:**
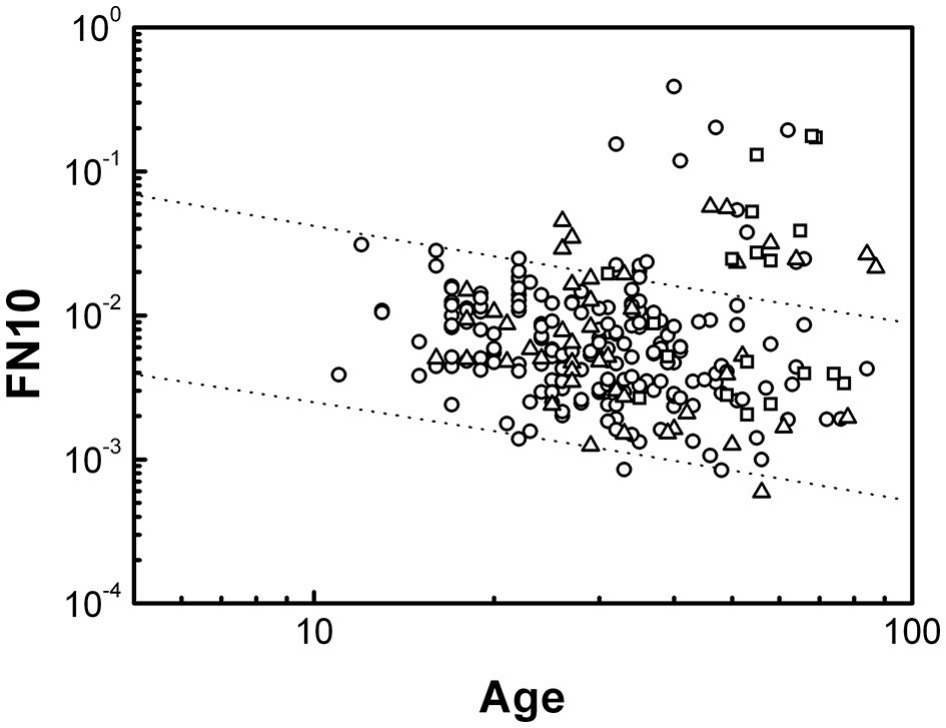
Dependence of FN10 on age expressed in years for individuals with CD. The lines represent the behavior determined in Figure 2 for healthy individuals. Data from the three groups are shown: GA (circles), GB (triangles), and GC (squares).

Figure 3 shows the values of FN10 in individuals with CD. Significant deviations are observed in the three groups GA, GB and GC, with data that deviate from the values predicted for a healthy individual beyond the prediction intervals. Furthermore, the deviations are toward higher values of FN10. Individuals with CD who present FN10 values with such deviations will be considered positive in FN10 (FN10^+^).

**Figure 4:**
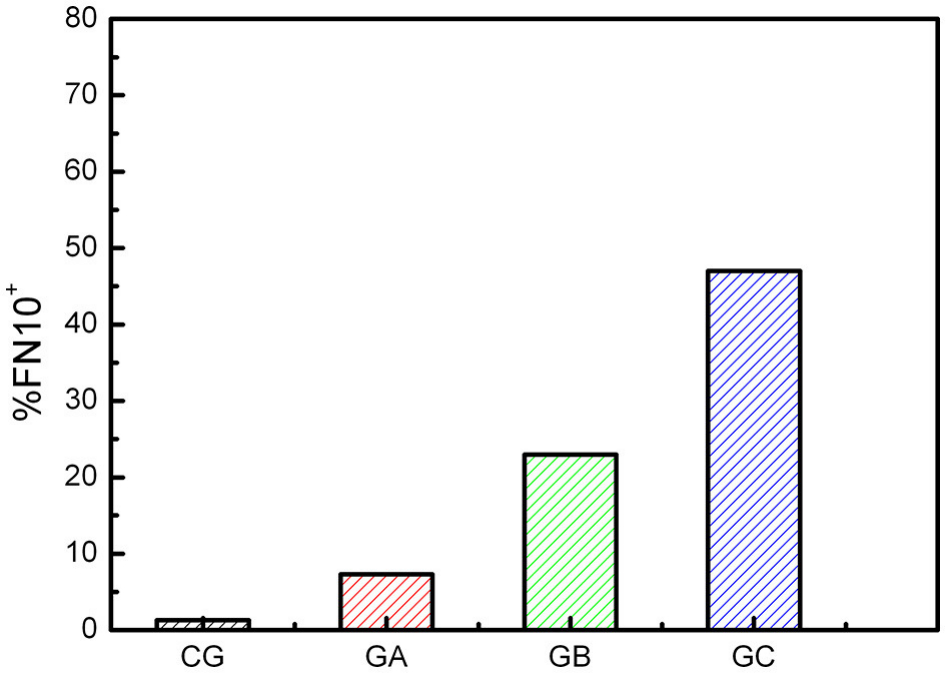
Percentage of FN10^+^ CD individuals for each group. There is a small percentage of false positives in the control group (CG).

Figure 4 shows the percentage of FN10+ individuals in each group. A progression is observed with the advancement of heart disease. In GA, GB, and GC there are individuals with normal FN10 values. This is expected because some electrical or echocardiographic abnormalities may not be serious enough to alter cardiac dynamics. Within each group there are no significant differences between sexes.

## 4 Discussion and conclusions

In this work, we use the FNN method to analyze HRV time series. This method is well known in the analysis of nonlinear signals and is based on the unfolding of the phase space using the time-delay reconstruction method (56, 58). Over years of research, very different methods have been used to study HRV. Initially, it was considered the result of the action of multiple biological clocks. Advances in invasive and noninvasive cardiophysiology made it possible to establish that HRV emerges from SAN. The ANS and SAN were then considered the main contributors to the HRV (20–22), and the VLF, LF, and HF bands were defined under the hypothesis that their contributions occur at different frequencies (4–6).

The most recent research presents a different scenario (48). While the ANS contributes nearly constant power over a wide frequency range, the contribution of the SAN begins at low frequencies and evolves with growth, spreading, and eventually occupying the full frequency space, and it even overlaps the peaks of respiratory sinus arrhythmia.

We established that the contribution of the SAN is related to its architecture and conjectured that the fibrotic process is mainly responsible for the changes observed in HRV in healthy subjects (48). Furthermore, HRV is transmitted unchanged from the SAN to the atrioventricular node (AVN), as revealed by the synchronization of the various intervals of the cardiac cycle (59). This transmission is possible due to the electrical insulation that the fibrotic tissue provides to the SAN. It has recently been established that fibrosis plays a fundamental role in the normal function of the SAN, regulating neuronal stimulation in the healthy heart (39, 60). Interstitial fibrosis is inherent to the SAN, providing structural support and electrical insulation, but also infiltrating it, thus determining its microstructure. Since fibrosis, which is detected even in fetuses as from the fifth month of gestation (61, 62), progresses continuously, it would be more appropriate to consider that the HRV signal has a continuous spectrum that evolves smoothly with growth.

The HRV has been described as a sum of correlated signals (or colored noises), to study its scaling properties. Colored noises are generated from white noise by limiting the power of each frequency. We prefer to avoid mentioning noise, in the sense that our correlated signals do not come from stochastic phenomena, but from very sensitive complex processes. The color of a signal is manifested in the shape of its FNN curve, with a white signal having a nearly flat FNN curve close to 1. A brown signal is more correlated than a pink one, and its FNN curve decreases faster, thus the latter has an intermediate behavior between white and brown.

The FNN curve reflects the color of the signal or its degree of correlation or complexity and can be used to indicate changes in the HRV due to growth or different pathologies (51). The FN10 index was defined because *d*_0_ = 10 is the highest embedding dimension that we find in healthy adults (49, 50). By definition, FN10 is a decreasing function with age at least for healthy individuals over 5 years of age.

In this work we determine that FN10 has a scaling behavior with age, which is independent of sex.

In Chagas disease, some individuals show FN10 values significantly above those seen in the healthy population, in a percentage that increases with the progression of the disease. Higher FN10 values indicate a loss of correlation (or loss of complexity) between heartbeats, which is a widely documented phenomenon in individuals with atrial and ventricular arrhythmia or fibrillation, and congestive heart failure (CHF). In individuals with CHF the loss of correlation is attributed to both reduced SAN activity and increased ANS regulation (20, 27, 28, 49, 50). In arrhythmias and fibrillation, the loss of complexity is attributed to unregulated muscular activity.

The fibrotic process can be present in the early stages of CD even in the absence of other clinical manifestations. The characteristic diffuse spread out of the fibrosis can alter the contribution of SAN by disordering its structure. Furthermore, the production of anti-*M*_2_R Ab, also detected in idiopathic heart failure, was documented in CD producing a deregulated contraction (not mediated by the ANS) of the cardiac muscle fibers (35). Inflammation and fibrosis in Chagas disease can occur at any site of the conduction system and are accompanied by the infiltration of lymphocytes, plasmocytes and macrophages. However, the relationship between antibodies and fibrosis has not yet been explored. All of these mechanisms lead to a loss of complexity in the HRV and higher FN10 values.

The main findings of our work are as follows:

(1) In healthy individuals FN10 has a decreasing scale behavior with age, independent of sex, according to the smooth changes in the frequency structure of the HRV.

(2) During Chagas disease, FN10 exhibits positive deviations that in the GB and GC are compatible with the pathophysiological mechanisms that determine the progression of the disease (mainly denervation and fibrosis). We conjecture that a similar deviation in GA may be an indicator of individuals with preclinical alterations of the disease.

Chronic CD evolves with variable prognosis among individuals. It constitutes a major health problem due to endemic conditions in Latin America, even though only a relatively small percentage of individuals with *T. cruzi* infection develop heart disease. Developing biomarkers to predict clinical outcomes is a recognized need to improve benznidazole treatment protocols, develop and evaluate new drugs, and study the role of other infections or non-communicable diseases in the treatment of individuals with chronic CD.

The results we present in this work indicate that FN10 may be a candidate prognostic biomarker for heart disease. FN10 is calculated from HRV time series. The calculation is robust in series without artifacts and with a length >20,000 beats. Furthermore, the calculation algorithm should be standardized. For its use in clinical practice, it would be necessary to implement a procedure to check the goodness of the calculation, for example, repeating the calculation with different segments of a 24-h HRV time series.

The main limitation of our study is its cross-sectional nature. Longitudinal studies could provide stronger evidence for FN10 as a prognostic biomarker, although performing them is beyond our capabilities. It would also be important to validate FN10 with an external cohort to establish the generalizability of our findings across different populations. In the population under study, we have not correlated the severity of the symptoms with the increase in FN10, though in previous studies we reported an increased FN10 in individuals with supraventricular and ventricular arrhythmia, and to CHF, as compared with healthy individuals (50).

We have work in progress assessing the correlation between FN10 and serum level of anti-*M*_2_R Ab and we are conducting a non-parametric frequency study on Chagas disease.

## Data Availability

The raw data supporting the conclusions of this article will be made available by the authors, without undue reservation.
